# Pattern search in BioPAX models

**DOI:** 10.1093/bioinformatics/btt539

**Published:** 2013-09-16

**Authors:** Özgün Babur, Bülent Arman Aksoy, Igor Rodchenkov, Selçuk Onur Sümer, Chris Sander, Emek Demir

**Affiliations:** ^1^Computational Biology Center, Memorial Sloan-Kettering Cancer Center, New York, NY 10065, USA, ^2^Tri-Institutional Training Program in Computational Biology and Medicine, New York, NY 10065, USA and ^3^Banting and Best Department of Medical Research, The Donnelly Centre for Cellular and Biomolecular Research, University of Toronto, Toronto, Ontario M5S 3E1, Canada

## Abstract

**Motivation:** BioPAX is a standard language for representing complex cellular processes, including metabolic networks, signal transduction and gene regulation. Owing to the inherent complexity of a BioPAX model, searching for a specific type of subnetwork can be non-trivial and difficult.

**Results:** We developed an open source and extensible framework for defining and searching graph patterns in BioPAX models. We demonstrate its use with a sample pattern that captures directed signaling relations between proteins. We provide search results for the pattern obtained from the Pathway Commons database and compare these results with the current data in signaling databases SPIKE and SignaLink. Results show that a pattern search in public pathway data can identify a substantial amount of signaling relations that do not exist in signaling databases.

**Availability:** BioPAX-pattern software was developed in Java. Source code and documentation is freely available at http://code.google.com/p/biopax-pattern under Lesser GNU Public License.

**Contact:** patternsearch@cbio.mskcc.org

**Supplementary information:** Supplementary data are available at *Bioinformatics* online.

## 1 INTRODUCTION

BioPAX is a community standard for pathway representation developed by a broad community of researchers working on pathways and related resources (Demir *et al.*, 2010). It can represent metabolic and signaling pathways, molecular and genetic interactions and gene regulation networks. Currently, there are >30 pathway resources that support BioPAX representation, listed in Pathguide (Bader *et al.*, 2006).

The detailed structure of BioPAX allows it to cover a wide spectrum of biological phenomena; however, its complexity also creates a significant barrier for researchers to effectively use it. Paxtools is a Java library that was developed to address this need. It exposes BioPAX models as Java objects and provides an array of utility methods (Demir *et al.*, 2013) to query its contents. Although Paxtools facilitates simple searches, coding more complex queries that require evaluating links between multiple objects can be tedious and error-prone. What is often needed is a way to efficiently define and run such queries as graph patterns.

Existing graph searching tools for biological networks are limited to simple binary graphs that use nodes for molecules and edges for the interactions between (Berg and Lässig, 2004; Ferro *et al.*, 2007; and Giugno and Shasha 2002). Generic RDF/OWL tools such as SPARQL and OWL-Reasoners allow defining more complex searches; thus, it is more suitable for searching rich BioPAX pathways. Unfortunately, there are still many biologically relevant patterns that cannot be captured by RDF/OWL tools (see Supplementary Data).

Here we present a framework and software tool that allows users to specify complex patterns using the rich BioPAX ontology and to search any BioPAX level 3 model for those patterns. We demonstrate the tool with a pattern sample that we query in Pathway Commons (Cerami *et al.*, 2011) database and provide the search result as Supplementary Data. The tool can be used as a standalone application or a library to seamlessly integrate pattern searches into the existing software.

## 2 METHODS

We define a pattern as a fixed number of BioPAX elements that satisfy a list of constraints. Constraints are re-usable objects that can be mapped to elements in the pattern.

**Fig. 1.**
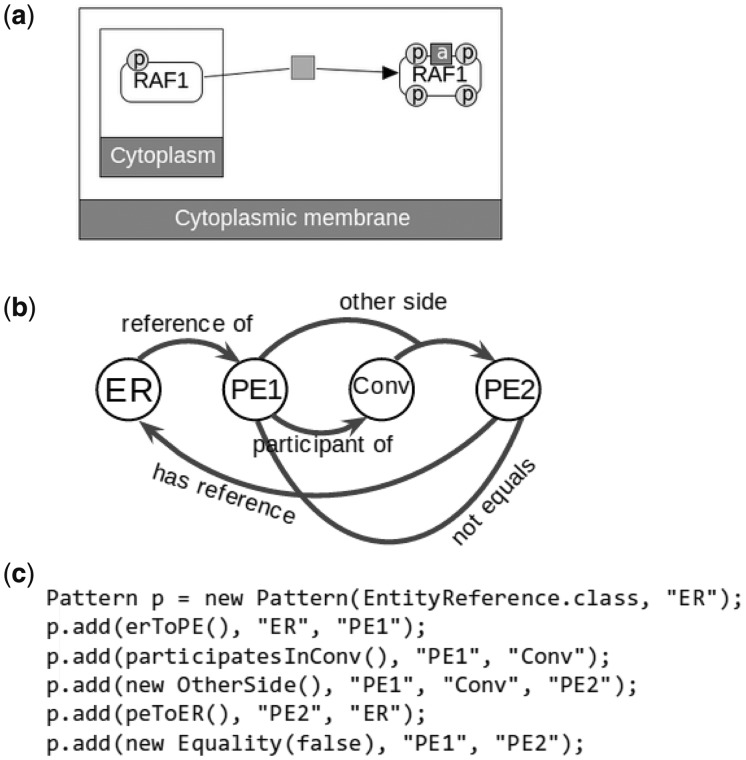
(**a**) Phosphorylation and activation of RAF1, drawn using ChiBE (Babur *et al.*, 2010). The gray rectangle represents the Conversion that modifies RAF1. (**b**) Visual representation of a pattern that captures modification process of a protein, where circles represent BioPAX objects, and arcs represent constraints. Each constraint is mapped to a fix number of BioPAX objects. (**c**) A part of the Java code that creates the illustrated pattern

Consider a case where we want to detect reactions that post-translationally modify a protein—an important question for proteomic data analysis. An example is shown in Figure 1a, where RAF1 is activated by phosphorylation and translocation. Figure 1b contains a diagram representing a sample pattern that captures such a relation. This pattern is composed of four BioPAX elements and five constraints. The pattern includes an *EntityReference* (ER), which has at least two different *PhysicalEntity* associated (PE1 and PE2) (*EntityReference* provides mapping to a specific entry in a reference molecule database, like UniProt. *PhysicalEntity*, on the other hand, is a specific modification state at a specific cellular location of that entity), and a *Conversion* (Conv) that has PE1 and PE2 as participants on different sides.

Constraints that can enumerate matching objects of last element for a given set of assigned prior elements are called *generative* constraints. For example, in Figure 1c, the first constraint can search for member *PhysicalEntity* objects, given that the ER is already assigned; thus, it is generative. However, the last constraint checks for inequality and needs both of its mapped elements already assigned to an object; thus, it is not generative.

### 2.1 Constructing and searching a pattern

To construct a pattern, users first should decide the types of objects in the element array and their relationships. The type of the first element should be specified explicitly. The types of the following elements are determined by the generative constraints. Every element in the pattern, other than the first one, should be generated with a distinct generative constraint. Currently, there is no look-ahead, in the sense that constraints cannot use elements that are supposed to be generated by the next constraints. For instance, the first constraint in Figure 1 generates PE1 from ER, and the second constraint generates Conv from PE1. We cannot swap their locations because the second constraint depends on PE1, which is generated by the first constraint. There is no limit for the number of non-generative constraints, but their position in the pattern should also satisfy dependency order.

The software provides a wide range of constraints, which can be classified as follows:

- *Simple link traversing constraints*: These can be any property linking two BioPAX elements, including transitive properties, such as a member of a complex with arbitrary depth.
- *Element field constraints*: Constraining element fields with either pre-set values or with values of other elements, such as two objects sharing or not sharing a GO term annotation.
- *Constraints for BioPAX semantics*: These constraints implement non-trivial reasoning, such as identifying inputs and outputs of a Conversion, linking entities through recursive homology relationships also taking complex memberships into account or detecting syntactically invalid structures like an input to a Conversion also being a controller.
- *Logical operator constraints*: Logical operators, like AND, OR and NOT, can be applied to constraints using those wrapper constraints.

Users can run a graph search using constructed patterns on any level 3 BioPAX model. Previous levels of BioPAX can be auto-converted to level 3 using Paxtools. Running times of the searches depend on the pattern to be searched. The search method is iterative, i.e. it evaluates candidate members for the slots in the pattern linearly and outputs every possible matching.

It is also possible to pre-assign some elements in the pattern to conduct more specific searches. An example is provided in the Supplementary Data. Patterns can be easily extended, combined and re-used, allowing users to define increasingly complex biological processes, such as signaling cascades as patterns, and to share them with other researchers.

## 3 RESULTS AND DISCUSSION

We demonstrate the use of pattern searches with a biologically interesting example in the Pathway Commons database, which is an aggregate of public pathway databases that provide their data in BioPAX. The example pattern detects pairs of molecules where the first one is controlling an interaction that the second one participates in. This is useful for capturing signal transduction in the cell. Unlike the simple example in Figure 1, this pattern also handles homology relations and molecular complexes. The pattern and the search results are given in the Supplementary Data.

Searching this pattern in Pathway Commons returns 32 599 relations between 4695 proteins. We compared these results with the relations that we could get from public signaling databases SPIKE (Paz *et al.*, 2011) and SignaLink (Fazekas *et al.*, 2013). Even though our results are comparable with other signaling databases in size, only ∼10% of data are overlapping (see Supplementary Data). This shows that there can be a lot to gain by searching patterns in public detailed models, even if there are databases specialized for the information of interest. The project Web site contains other ‘how to’ examples and documentation.

*Funding*: This research was supported by NIH grants (U41HG006623) and (GM103504).

*Conflict of Interest*: none declared.

## Supplementary Material

Supplementary Data
