# Supplementary material for: Pattern search in BioPAX models
Source: Bioinformatics. 2013 Sep 16;30(1):139–40. doi: 10.1093/bioinformatics/btt539 (PMC3866551; doi:10.1093/bioinformatics/btt539)
Supplement: Supplementary Data [file supp_30_1_139__index.html]

Pattern search in BioPAX models — Pattern search in BioPAX models — Supplementary Data 

# Pattern search in BioPAX models

## Supplementary Data

files

**Files in this Data Supplement:**

- Supplementary Data - pdf file
- Supplementary Data - txt file
