# Supplementary material for: Pattern search in BioPAX models
Source: Bioinformatics. 2013 Sep 16;30(1):139–40. doi: 10.1093/bioinformatics/btt539 (PMC3866551; doi:10.1093/bioinformatics/btt539)
Supplement: Supplementary Data [file supp_btt539_Suppl.Doc.pdf]

Pattern search in BioPAX models

Supplementary data

A sample pattern

Here we describe a pattern sample for BioPAX models defined using the pattern search framework described in the manuscript. This pattern captures directed relations between pair of proteins, where the first protein controls an interaction that the second protein participates in. The aim of this pattern is to detect units of signaling in the cell with upstream and downstream proteins identified. The pattern makes sure that the controller protein is not also a participant of the interaction.

Illustration

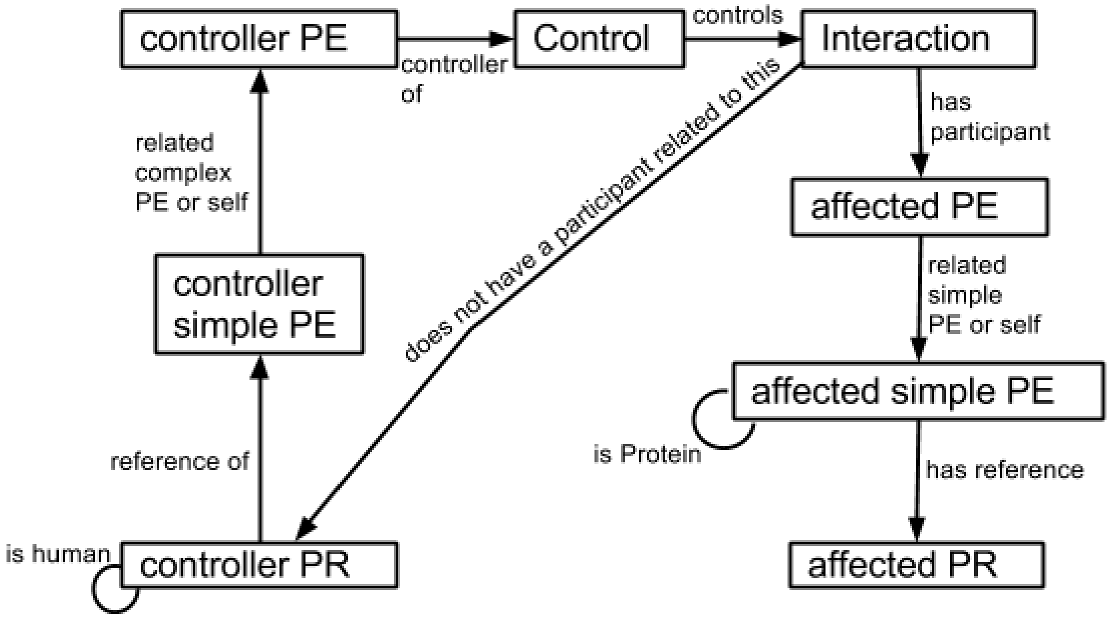

In the element names, PE is used for indicating a `PhysicalEntity`, and PR is used for indicating a `ProteinReference`. A "simple PE" refers to a `PhysicalEntity` that is not a `Complex`, and has an associated `EntityReference`, whereas other PE can be a `Complex`, or a generic homology node that does not have an associated `EntityReference`. Note that the homology and complex memberships can be arbitrarily nested in BioPAX, and this pattern captures all necessary connections between them.

Java code

```
import org.biopax.paxtools.model.level3.Protein;
import org.biopax.paxtools.model.level3.ProteinReference;
```

```java
import org.biopax.paxtools.pattern.Pattern;
import org.biopax.paxtools.pattern.constraint.*;
import static org.biopax.paxtools.pattern.constraint.ConBox.*;
…
// Start with the reference of the controller protein.
Pattern p = new Pattern(ProteinReference.class, "controller PR");

// We are interested in human data.
p.add(isHuman(), "controller PR");

// Get to a PhysicalEntity of the controller protein.
p.add(erToPE(), "controller PR", "controller simple PE");

// Allow generic entities and complexes in the pattern. “controller PE” can be a parent
// generic entity, or a parent complex of “controller simple PE”. These two objects can
// also be equal, so involvement of the complex structure is not necessary to detect the
// pattern.
p.add(linkToComplex(), "controller simple PE", "controller PE");

// Get to the Control object that the first protein is a controller.
p.add(peToControl(), "controller PE", "Control");

// Get to the interaction controlled by the Control.
p.add(controlToInter(), "Control", "Interaction");

// Make sure that none of the participants of the Interaction is related to the
// controller protein.
p.add(new NOT(participantER()), "Interaction", "controller PR");

// Get to an affected participant of the Interaction
p.add(participant(), "Interaction", "affected PE");

// If the affected participant is a generic or complex node, get to the simple
// EntityReference. “affected PE” and “affected simple PE” can be equal, so that the
// pattern can also match to simple structures.
p.add(linkToSimple(), "affected PE", "affected simple PE");

// Make sure that the affected molecule is a Protein.
p.add(new Type(Protein.class), "affected simple PE");

// Get to the reference of the affected protein.
p.add(peToER(), "affected simple PE", "affected PR");
```

Running time

Running time of pattern search depends on the structure of the pattern. It also depends on the structure of the BioPAX model. The search of this sample pattern traverses through controlled interactions and its participants. It also traverses through homology and complex memberships. In the worst case, where every protein is a parent generic molecule of all other proteins, and all pairs of proteins are connected through an interaction, the search would be infeasible due to long running time. However, this is not the case in current biological models. Proteins generally have a few generic parents, if any. Number of interactions controlled and participated are generally low for most proteins, while it can be high for hub proteins. Using the *O*-notation, we can say that the running time is bounded by *O(kn)*, where *n* is the number of proteins, and *k* is the average number of other proteins that can be reached through a controlled interaction. We observe that *k* is around 12 in Pathway Commons database.

Results

Searching this pattern in Pathway Commons database takes under 1 minute using a PC with 3.4 GHz processor and 8GB of RAM, and returns 32599 matches with 4695 proteins that have an associated HGNC symbol. These relations are given in the supplementary data file “directed-relations.txt”. Below are three example matches, whose details are illustrated with ChiBE.

ZDHHC21 --> NOS3

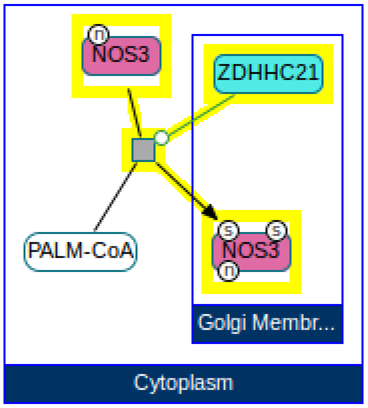

IL12A --> JAK2

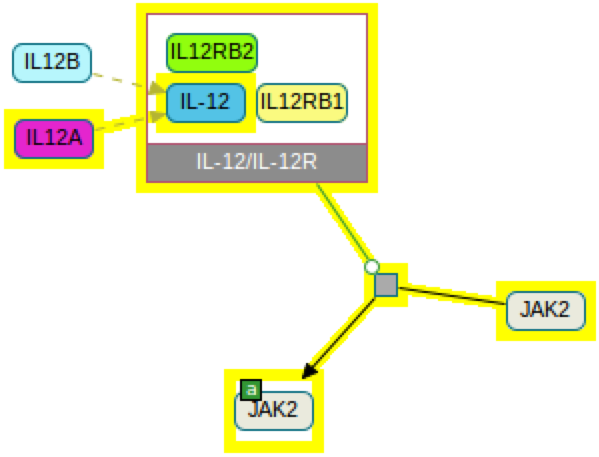

NTRK1 --> SHC3

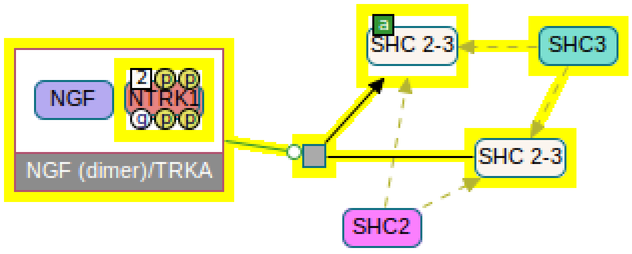

These results are a ready-to-use resource to study signaling in the cell. We compared these to the two major signaling databases SPIKE and SignaLink. Please see the next section for the information on how we parsed those databases. SPIKE contains 17686 directed relations, and SignaLink contains 37564 directed relations that we could map participating entities to HGNC symbols. Below diagram shows the intersection between these resources.

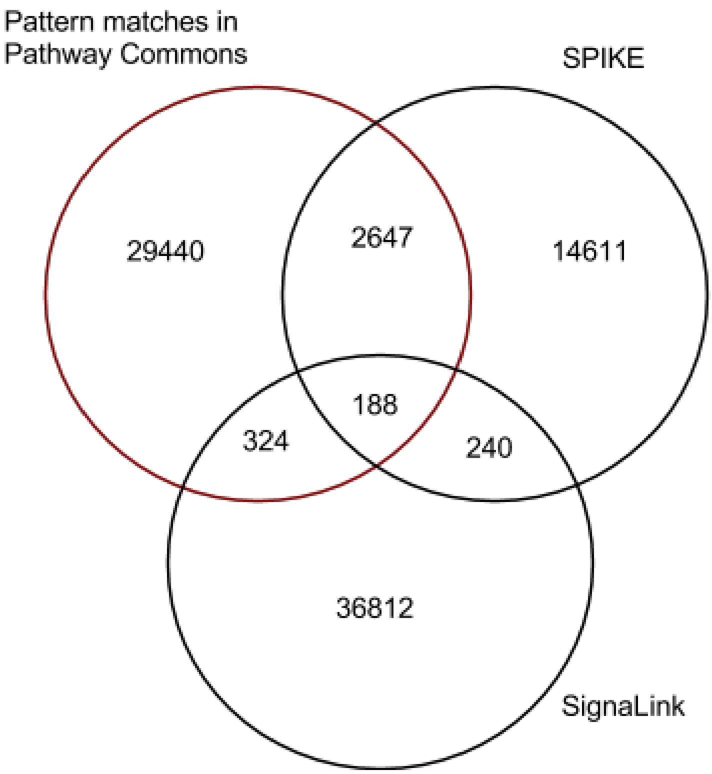

SignaLink is dominated by inferred relations, while SPIKE and Pathway Commons only include data from curated resources. That partly explains the very low overlap of SignaLink with others. Our pattern search result covers about 17% of SPIKE relations, and it is larger in size. Also note that none of the three example matches above are covered by SPIKE or SignaLink.

Here, we demonstrate that there is a lot to gain by mining patterns in detailed pathway models. Searching this example pattern in Pathway Commons generates a resource that is as valuable as other signaling databases, with many uniquely covered relations.

Parsing SPIKE and SignLink

We parsed XML data of SPIKE from http://www.cs.tau.ac.il/~spike/formats.html on July 19, 2013. We iterated over “Regulation” elements whose “Source” and “PhysicalTarget” properties are mapped to “Gene” or “Group” elements. Regulation elements contain information of directed relations. We parsed contents of the source and targets to get Entrez Gene IDs of the regulators and targets, then mapped these to HGNC symbols.

We parsed SignaLink from http://signalink.org/download using the “complete database” option on July 19, 2013. SignaLink provide their complete data as an SQL database. We used the following SQL query to generate a file of directed binary relations between gene names:

```
select n11.name, n22.name
into outfile "/tmp/signalink.txt"
from names n1, names n11, names n2, names n22, interaction i
where i.is_directed = 1
and i.source = n1.id
and n1.protein_id = n11.protein_id
and n11.type="GN"
and i.target = n2.id
and n2.protein_id = n22.protein_id
and n22.type = "GN";
```

Genes have multiple names in the SignaLink database. We further filtered the query result by selecting pairs that contain HGNC symbols as names for both proteins.

Pre-assigning elements of a pattern for a search

Pattern searches can be made more specific by pre-assigning some of the elements of the pattern. The example below pre-assigns the homology entity representing activated ERK proteins to the “controller PE” element of the sample pattern so that the search only retrieves downstream of the activated ERK.

```
// Assume we use the same sample pattern.
Pattern pattern = …

// Assume we already have a model loaded.
Model model = …

// We get the PhysicalEntity representing the homology entity that represent activated
// ERKs using its ID. This could be the result of a name search, or any other kind of
// computation. Name of this entity is “ERK1-2-active”.
PhysicalEntity erk = (PhysicalEntity) model.getByID(
       "http://pid.nci.nih.gov/biopaxpid_4936");

// Initiate a new empty pattern match.
Match m = new Match(pattern.size());

// Set erk as “controller PE”.
m.set(erk, p.indexOf("controller PE"));

// Search the model using the pre-assigned Match object.
List<Match> result = Searcher.search(m, p);
```

Using BioPAX-pattern with minimal programming effort

BioPAX-pattern is designed primarily to be used as a library, however, it is also possible to use it without any programming. The framework includes a GUI which lets users to select a predefined pattern of interest, a resource model to search, and name of output file. The GUI can be launched by directly executing the distributed jar file.

```
java -Xmx5G -jar biopax-pattern.jar
```

Note that the jar filename can be different to include the version of the release. Above call launches the below dialog.

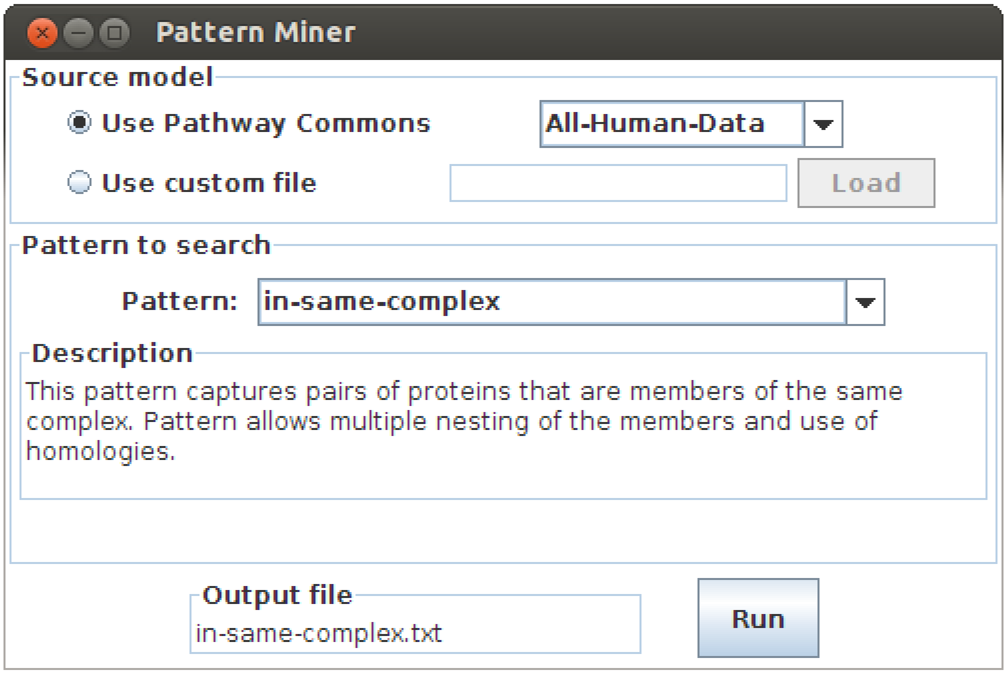

This dialog also helps users to do minimum programming to search for a pattern that they define. In the below example, we define a `Miner` object, which knows the pattern and how to write the results to a text file. Then we use the same GUI to use this miner.

```
Miner miner = new MinerAdapter("name-of-the-pattern", "Description of the pattern")
{
    public Pattern constructPattern()
    {
        Pattern p = … // Construct the pattern here.
    }
```

```
    public void writeResult(Map<BioPAXElement, List<Match>> matches,
        OutputStream out) throws IOException
    {
        // Write text results to the provided output stream here. Typically, iterate
        // over the result Match objects and write IDs or names of the elements of
        // interest in the Match.
    }
};

// Launch the GUI that will assist choosing a source model, and output file name.
Dialog d = new Dialog(miner);
d.setVisible(true);
```

Other software that process BioPAX

BioPAX-pattern is currently the only software that facilitates defining and searching patterns in a BioPAX model. It uses Paxtools for reading BioPAX into memory.

Instead of using a dedicated software, generic RDF/OWL tools such as SPARQL and OWL-Reasoners can also be used to search patterns in BioPAX. Although these tools can cover some of the same use cases and also can be used for situations where BioPAX is used in combination with other RDF data, they have two problems:

(i) Generic tools can not handle certain BioPAX semantics. During the development of BioPAX, the community discovered limitations of OWL. As a result, not all of the biological semantics were captured using the formal specification and were only mentioned in the documentation. It is difficult and/or impossible to search for these using SPARQL or OWL reasoners.

An example pattern is finding reactions that are “entity-balanced for proteins”, where for each protein participant on the left side there is a corresponding protein on the right side that belong to the same ProteinReference. This is an important question for finding curation errors and separating degradation and translation events from other protein modification events. This matching is further complicated by stoichiometry, complexes and generics. For example, consider a reaction where a homotrimer protein complex, upon phosphorylation of one of its components, disassociates into a phosphorylated monomer and a homodimer complex. This reaction is still considered entity-balanced. It is not possible, however, to define satisfiability of such bijective functions as constraints in SPARQL or any OWL reasoner that we are aware of. It is possible to define them as pattern constraints in our framework.

(ii) Paxtools is the most widely used library for developing software that processes BioPAX. Using RDF/OWL tools together with Paxtools requires developers to load their model into the memory as a separate instance or use a triple store in parallel to the Paxtools, run the search in an external tool, and then retrieve the subnetworks and load them back to Paxtools.

This process can be prohibitively slow, especially for large models.

Below, we list other software that can process BioPAX.

Paxtools: Java library for reading and writing BioPAX. The core module provides a Java object model, accessor methods, and a hibernate binding. The library contains various modules for different tasks, such as graph theoretic-querying module, BioPAX to SBGN-ML conversion module, PSI-MI to BioPAX conversion module, BioPAX to gene set conversion module, and binary interaction module.

BioPAX Validator: BioPAX Level 3 data validation. Uses a community-defined set of rules and best practices to detect errors and warnings in a BioPAx model. Uses Paxtools for reading BioPAX.

ChiBE: Pathway visualization software for BioPAX models. Uses Paxtools to load a BioPAX model, then creates a view similar to SBGN-PD.

Cytoscape: Pathway visualization tool. Includes a BioPAX reader.

BiNoM: A Cytoscape plugin for network analysis. Includes a reader and writer for BioPAX Level 3.

Systems Biology Linker (Sybil): An application that can visualize BioPAX and convert it to SBML. This application is a part of the Virtual Cell environment.

rBiopaxParser: A tool for parsing, viewing and modifying BioPAX pathway data within the R environment.

A more comprehensive and dynamic list of BioPAX software can be found at:
http://sourceforge.net/apps/mediawiki/biopax/index.php?title=Biological_software_supporting_BioPAX
